# RGB Magnetophotonic Crystals for High-contrast Magnetooptical Spatial Light Modulators

**DOI:** 10.1038/s41598-018-37317-9

**Published:** 2019-01-24

**Authors:** Soheila Kharratian, Hakan Urey, Mehmet C. Onbaşlı

**Affiliations:** 1 Department of Materials Science and Engineering, Koç University, Sarıyer, Istanbul 34450 Turkey; 2 Department of Electrical and Electronics Engineering, Koç University, Sarıyer, Istanbul 34450 Turkey

## Abstract

Magnetooptical spatial light modulators (MOSLMs) are photonic devices that encode information in photonic waveforms by changing their amplitude and phase using magnetooptical Faraday or Kerr rotation. Despite the progress on both MO materials and switching methods, significant improvements on materials engineering and SLM design are needed for demonstrating low-power, multicolor, analog and high-contrast MOSLM devices. In this study, we present design rules and example designs for a high-contrast and large figure-of-merit MOSLM using three-color magnetophotonic crystals (MPC). We demonstrate for the first time, a three-defect MPC capable of simultaneously enhancing Faraday rotation, and high-contrast modulation at three fundamental wavelengths of red, green and blue (RGB) within the same pixel. We show using 2D finite-difference time-domain simulations that bismuth-substituted yttrium iron garnet films are promising for low-loss and high Faraday rotation MOSLM device in the visible band. Faraday rotation and loss spectra as well as figure-of-merit values are calculated for different magnetophotonic crystals of the form (H/L)^p^/(D/L)^q^/(H/L)^p^. After an optimization of layer thicknesses and MPC configuration, Faraday rotation values were found to be between 20–55° for losses below 20 dB in an overall thickness less than 1.5 µm including three submicron garnet defect layers. The experimental demonstration of our proposed 3-color MOSLM devices can enable bistable photonic projectors, holographic displays, indoor visible light communication devices, photonic beamforming for 5 G telecommunications and beyond.

## Introduction

Spatial light modulator (SLM) is an optical device, which uses an array of pixels to control the amplitude and phase of light as a function of position. Magnetooptical spatial light modulators (MOSLMs) use magnetooptical Faraday or Kerr effect to rotate the polarization of light to toggle between different polarization angles. By using polarizers to selectively filter or let out optical signals at different polarization angles, one can achieve analog modulation of light. Pixels can be controlled by reversing magnetization states of MO materials in MOSLMs. Magnetization reversal on a pixel level also reverses the magnetooptical Faraday and Kerr rotations of the pixels and these effects are observed in the output beam intensity^1–3^.

MOSLMs hold promise for emerging device applications because these nonvolatile devices enable ultrafast modulation and steering of optical beams due to the intrinsic high speed of magnetization reversal process, which can be tuned from nanoseconds^4–6^ down to a few tens of femtosecond time scales^7^. Magnetization reversal-based spatial light modulation may help span the potential modulation frequency range from DC to GHz (down to 1 THz) rates, enabling ultra-broadband optical telecommunication. Employing magnetism in SLM technology also offers the possibility of using thin films and reducing pixel size. With the recent demonstrations of voltage or current control of magnetization reversal^8–10^, MOSLMs can turn into integrated devices. Moreover, MOSLM is a robust solid-state device without mechanically moving parts, that functions also as a memory element due to the remanent magnetization and nonzero coercivity^11,12^. These features make MOSLMs useful for various applications such as optical circulators and isolators^13^, photonic projectors^14^, optical beamforming for visible light communication devices^15^, beam steering devices for various imaging and display technologies^16^, and more importantly holographic applications and 3D displays where high spatial resolution and short response time are crucial^17–19^. Previous demonstrations of SLMs other than MOSLMs include liquid crystal-based devices (LCD and LCOS) and digital micro-mirror devices (DMDs) with pixel sizes about 5 and 10 µm and response times in the order of 100 and 10 µs, respectively^20^. These SLMs are well-established products being used for many applications, however, they do not provide fast modulation and high resolution simultaneously. MOSLMs on the other hand, showing the potential for this requirement, have been extensively studied in the last decades but have not been successfully industrialized yet due to the accompanying challenges that necessitates more research in this area.

MOSLM suffers from high power consumption originating from large magnetic field needed to switch the pixels and high optical losses of MO materials. This causes Joule heating and consequently switching errors in current-driven MOSLMs^2^. Many studies were done in order to reduce power consumption by engineering the device configuration and drive lines^3,12^ or material and synthesis conditions^21,22^, and voltage-driven MOSLM was proposed by Park *et al*.^23^ to alleviate power dissipation and heating problem of drive lines. Another challenge with MOSLMs is the inherent weakness of MO effects especially in visible band, while pixel contrast in these modulators depends on the MO rotation angle. Kerr effect is generally very small so we focus on Faraday effect in this study. Magnitude of Faraday rotation (FR) depends on the optical path length of light in the MO material. Therefore, in order to realize practical miniaturized and integrated devices for display and 3D imaging, enhancement of FR in thin MO films along with controlling optical loss in fundamental red, green and blue (RGB) wavelengths is essential^24–26^.

Hybridizing plasmonics with MO materials is one of the approaches that has drawn attention for enhancing the Faraday effect by achieving highly-localized field intensities due to surface plasmon resonances^27–33^. Chin *et al*.^33^ showed plasmonic enhancement of Faraday rotation up to one order of magnitude, however, it was not in the visible range and due to the small thickness of the MO film, the maximum FR obtained was only 0.80° that is not sufficient for device applications. Enhancement of field and consequently FR in this approach occurs in the length scales smaller than diffraction limit (λ_0_/2n) around the plasmonic structure^34^, limiting the net enhancement of FR. Therefore, in order to realize enhancement all over a thicker film and achieve a large final rotation angle, multiple layers of plasmonic arrays in the film are going to be needed^35^. This makes the fabrication very difficult and transmittance would significantly drop due to multiple layers of metallic arrays.

Another approach used to increase FR in thin films is magnetophotonic crystal (MPC)^14,36–43^. In this approach, the MO film is sandwiched between periodic dielectric layers of different refractive index (Bragg mirrors) and plays the role of an optical cavity for the photonic crystal. Such a configuration demonstrates a photonic band gap since continuous translational symmetry is broken and only discrete translational symmetry is found in periodic layers. In addition to the photonic band gap, the addition of dielectric defects further breaks this discrete translational symmetry, leading to sharp transmission peaks within the photonic band gap. Once these dielectric defect layers are also magnetooptical (i.e. the off-diagonal elements in their permittivity tensors are no longer zero), the time-reversal symmetry of the photonic band structure is also broken: ω(k) ≠ ω(−k)^44^. In such structures, once the phase matching condition is satisfied for a given wavevector of light, photonic crystal cavity modes can enhance the optical path length of light passing through the magnetooptical defect layer. Consequently, Faraday rotation is accumulated and enhanced with each pass. Inoue *et al*.^36^ showed that enhancement of FR is directly related to the localization of light in the MO layers. In a similar method, Moccia *et al*.^45^ demonstrated 12° of FR in a tri-layer of dielectric and MO metals. In addition to the considerable improvement of FR, this approach has the advantage of filtering the undesired light coming from a broadband source. Higher localization and consequently higher FR is predicted by increasing the number of mirror layers. One disadvantage of this approach is the accumulation of optical loss due to enhanced optical path length within the cavity. While Faraday rotation can be enhanced through higher number of MO cavity layers and cavity quality factor, in order to keep losses below an acceptable threshold, cavity layer count must be optimized. Furthermore, thickness, relative position and material properties of MO defects can vary in the MPC structure and the transmission spectra are also sensitive for such cavity and material parameters. Therefore, an optimization of the configuration and constituent materials is necessary to have an efficient device with high FR and low optical loss. In this study, we investigate properties of magnetophotonic crystals with different configurations, compare their figures of merit and propose a magnetooptical SLM which can perform efficient modulation with rotations higher than 20° in RGB wavelength. In the next sections, we first present the proposed device structure and its operation principle. Next, we evaluate the available MO material options based on their FR and optical loss in visible. Finally, design principles for RGB MOSLMs are presented.

## Proposed Device Structure and Operation Principle

**Figure 1:**
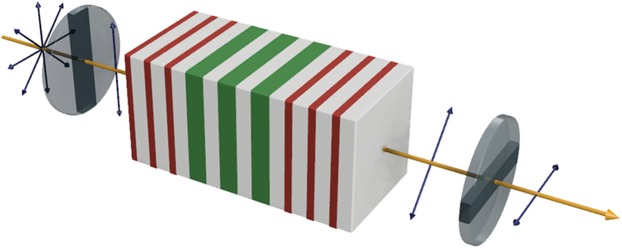
Schematic of proposed structure and operation principle for the magnetophotonic crystal-magnetooptical spatial light modulator (MPC-MOSLM) in transmission mode. A laser beam or light emitting diode optical signal with initial random polarization is incident on a polarizer. A vertically polarized light is incident on the magnetophotonic crystal structure in the format of (H/L)^p^/(D/L)^q^/(H/L)^p^ engineered to match maximum transmission and Faraday rotations in targeted wavelengths. The transmitted light acquires magnetooptical Faraday rotation and this polarization rotation is detected using an analyzer or a polarizer with horizontal orientation. The cavity structure consists of high- (red) and low-index (gray) layers as well as magnetooptical defect (green) layers. Upon reversal of the magnetization states of the magnetooptical layers, Faraday rotation is also reversed and since only one polarization component can pass through the output polarizer, partial or complete blocking of output signal occurs depending on the rotation angle.

The schematic structure of a transmission-type magnetophotonic crystal-magnetooptical spatial light modulator (MPC-MOSLM) is presented in Fig. 1. A randomly polarized wave coming from a light source becomes linearly polarized after passing through the first polarizer and propagates along the MPC. Polarization plane rotates due to Faraday Effect and the beam is analyzed by the second polarizer which is orthogonal to the first one. Only one polarization component may pass through the combination of polarizer and analyzer. By simultaneously controlling the magnetization in the defect layers of the MOSLM, the Faraday rotation of each defect layer can be switched from 0 to θ_F_ (Faraday rotation angle). Thus, depending on the degree of rotation, the output light intensity can be controlled. The full range of Faraday rotation angles are ±θ_F_ or 2θ_F_, but since the proposed structures here are optimized for three colors (red, green and blue) and their Faraday rotation angles are different, a common baseline of 0° rotation for each color must be used. By using the same 0° rotation angle baseline for all three colors and operating the MOSLM between 0 to θ_F_, the contrast values for all colors are maximized and a simple operation scheme is achieved.

In this design, the MOSLM pixel contrast is defined as the ratio of signal intensity levels between 0 and the transmitted light. This definition takes into account the fact that the baseline (0 signal) can be small and nonzero due to a small and unintentional angle between the polarizers at extinction condition (perpendicular orientations). By calling this angle θ_P_ (polarizer misorientation), we quantify the signal-to-noise ratio or MOSLM contrast as:

1 $${\rm{\Delta }}=\frac{\tan ({{\rm{\theta }}}_{{\rm{F}}})}{\tan ({{\rm{\theta }}}_{{\rm{P}}})}$$

where θ_P_ can be made arbitrarily small through an iterative feedback-controlled zeroing procedure for polarizer angles. Misalignment angles as small as 0.086° have been reported^46^ and we use this value to calculate contrast for the proposed MOSLM.

Both high FR and low loss are important factors for the MOSLM to realize high contrast and low power consumption, hence we define MO figure of merit of as:

2 $${\rm{FoM}}\,(^\circ \cdot {{\rm{dB}}}^{-1})=\frac{{\rm{Faraday}}\,\mathrm{rotation}\,(^\circ )}{{\rm{Optical}}\,\mathrm{loss}\,\,({\rm{dB}})}$$

We propose MPCs composed of TiO_2_ as high-index (H) and SiO_2_ as low-index (L) dielectrics for Bragg mirrors, and Bi_1_Y_2_Fe_5_O_12_ as MO defect (D), in the form of (H/L)^p^/(D/L)^q^/(H/L)^p^. TiO_2_ and SiO_2_ are highly transparent and provide adequate index contrast for a wide photonic band gap in visible range^47–49^. Comparing FoM calculated for different MO materials (Table 1), we chose bismuth-substituted yttrium iron garnet with composition of Bi_1_Y_2_Fe_5_O_12_ due to its higher figure-of-merit in RGB bands. Thicknesses of layers were initially chosen to be quarter-wave length for mirror dielectrics and half-wave length for MO films, however, for MPCs having more than one defect, an optimization process is necessary in order to render the transmission peaks at the desired wavelengths because the peak position is always affected by the presence of other peaks due to hybridization of magnetophotonic modes.

**Table 1:** Figure of merit (FoM) values calculated for different magnetooptical (MO) materials based on the values reported in the literature.

Material	Blue (474 nm)		Green (520 nm)		Red (633 nm)		Ref.
	Reported value	Calculated FoM value	Reported value	Calculated FoM value	Reported value	Calculated FoM value	
Y_3_Fe_5_O_12_	ε_xx_= 5.94 + 0.15060i ε_xy_ = 0.00801 − 0.00251i	0.24°/dB	ε_xx_ = 5.52 + 0.02410i ε_xy_ = 0.00036 + 0.00012i	0.09°/dB	ε_xx_ = 5.34 + 0i ε_xy_ = 0.00084 + 0.00084i	0.08°/dB	^52^
**Bi**_**1**_ **Y**_**2**_ **Fe**_**5**_ **O**_**12**_	**ε**_**xx**_ ** = 7.19 + 0.55723i** **ε**_**xy**_** = −0.06631** − **0.14457i**	**1.45°/dB**	**ε**_**xx**_ ** = 6.64 + 0.03313i** **ε**_**xy**_** = −0.07499** − **0.00634i**	**9.62°/dB**	**ε**_**xx**_ ** = 6.04 + 0i** **ε**_**xy**_ ** = −0.01697 + 0.00176i**	**1.18°/dB**	^52^
NiFe_2_O_4_	n = 2.53 + 0.79059i ε_xy_ = 0.01326−0.01889i	0.04°/dB	n = 2.62 + 0.68830i ε_xy_ = −0.00072 − 0.00923i	0.02°/dB	n = 2.58 + 0.38044i ε_xy_ = −0.00077 − 0.00005i	0.002°/dB	^64^
(BiDy)_3_(FeGa)_5_O_12_	ε_xx_ = 6.96 + 1.290393i ε_xy_ = −0.06934 − 0.00559i	0.35°/dB	ε_xx_ = 5.79 + 0.51333i ε_xy_ = 0.00020 + 0.01680i	0.19°/dB	ε_xx_ = 5.20 + 0.09728i ε_xy_ = 0.01164 − 0.00233i	0.51°/dB	^65^
Lu_2.5_Bi_0.5_Fe_5_O_12_	ε_xx_ = 6.88 + 0.60002i ε_xy_ = 0.00588 − 0.05661i	0.55°/dB	ε_xx_ = 6.43 + 0.19270i ε_xy_ = −0.01176 + 0.01342i	0.41°/dB	ε_xx_ = 5.78 + 0.02631i ε_xy_ = −0.00323 + 0.00506i	0.02°/dB	^66^
Lu_2.3_Bi_0.7_Fe_4.4_ Ga_0.6_O_12_	ε_xx_ = 6.48 + 0.44424i ε_xy_ = −0.00323 − 0.07393i	0.90°/dB	ε_xx_ = 6.17 + 0.16254i ε_xy_ = −0.02049 − 0.01245i	0.49°/dB	ε_xx_ = 5.62 + 0.02133i ε_xy_ = −0.00607 + 0.00370i	0.21°/dB	^66^
Gd_1.24_Pr_0.48_Bi_1.01_ Lu_0.27_Fe_4.38_Al_0.6_O_12_	ε_xx_ = 7.52 + 0.96644i ε_xy_ = 0.13229 − 0.05390i	0.91°/dB	ε_xx_ = 6.82 + 0.12387i ε_xy_ = 0.01803 − 0.05864i	2.88°/dB	ε_xx_ = 6.06 + 0.03278i ε_xy_ = −0.00130 − 0.01759i	1.15°/dB	^67^
Ce_1_Y_2_Fe_5_O_12_	FoM = 0.12°/dB	0.12°/dB	FoM = 0.096°/dB	0.096°/dB	FoM = 0.16°/dB	0.16°/dB	^50^

These oxides are candidates for the MO defect layers of the MOSLM device structure. The MO defect layer materials have been screened based on their MO FoM, which is defined as Faraday rotation (°) per optical loss (dB) for a single isolated slab of the material. Bi_1_Y_2_Fe_5_O_12_ (Bi:YIG), which is highlighted in Bold, has been found to have the highest overall FoM across visible wavelengths. For the rest of the simulations in this study, the magnetooptical properties of this Bi:YIG is used.

The necessary requirements of ideal MO materials for MOSLMs with imaging and display applications include (i) a wide material bandgap, (ii) low optical loss (i.e. optimal stoichiometry and minimum concentration of oxygen vacancies), (iii) stable structure, (iv) high Faraday rotation in the visible band. Doped garnets such as Ce:YIG (Ce_1_Y_2_Fe_5_O_12_) have been studied extensively for near infrared telecommunication. Although their FR are high (a few degrees per µm film), their losses dominate in the visible range and reduce the overall MO figure-of-merit^13,50,51^. For device fabrication purposes, novel high figure-of-merit MO material options also need to be developed.

Magnetic garnets generally have better optical and magnetooptical properties when epitaxially grown on single-crystal garnet substrates but we have used the experimental data of polycrystalline Bi_1_Y_2_Fe_5_O_12_^52^ for the simulations in this paper to stay realistic about the fabrication challenges. Therefore, epitaxial films are not essential and polycrystalline films should be sufficient for implementing the magnetophotonic structures presented in this study.

Previous efforts for growth of magnetic garnets on non-garnet substrates^13,53–56^ and realization of magnetophotonic crystals^37,43,57–59^ have mostly reported polycrystalline structure of the magnetic garnet. Growing magnetooptical iron garnet thin films on SiO_2_ or non-garnet layers may lead to lower Faraday rotation and slightly higher loss due to grain boundary scattering and imperfect control of crystalline orientation and stoichiometry with respect to epitaxial cases. A seeded-growth technique described in ref.^13,60^ helps alleviate this problem especially in the garnet thickness ranges used in this study. In the mentioned studies, seed layer or thermal annealing was used for fabrication of polycrystalline garnet on non-garnet substrates and no cracks or mechanical failure were observed for the thicknesses investigated, which are also close to the thickness ranges used in this study. In addition, as garnet thin film grains sizes on non-garnet layers may be larger than 5 µm in diameter, one may also use a seeded-growth and annealing approach for achieving near single crystal-quality small garnet pixel layers^61^. In order to preserve interface quality in the photonic multilayers, interdiffusion of layers must be avoided by growing the films at low enough temperatures in a pulsed laser deposition (PLD) or a sputtering chamber. After growth, thermal annealing^37^ has been shown to help crystallize the multilayers. Specifically for Bismuth-doped garnet films, due to the volatility of Bismuth, a sputtering or a PLD target rich in Bismuth must be used and the process temperature, growth rate, target-substrate distance as well as deposition pressures (base, *in situ* partial oxygen pressure) should be optimized for good structural, optical and magnetooptical quality.

In the following section, we investigate the performance of MPCs with different cavity structures and present an optimized RGB MPC for MOSLM device. We also illustrate how changes in the structure and material properties affect the behavior of such an MPC and device FoM.

## Results and Discussion

### One-defect MPC

**Figure 2:**
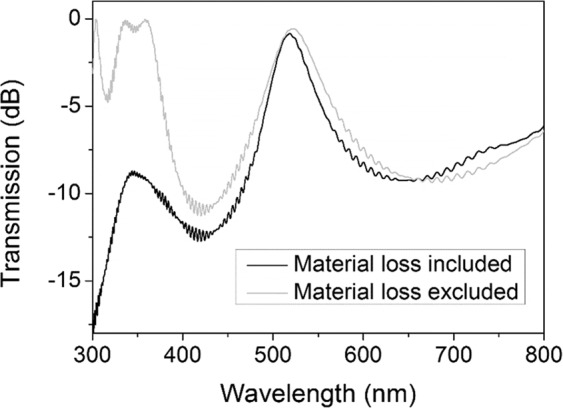
Transmission spectrum of the MPC with (H/L)/(D/L)/(H/L) structure. Black line shows the case where material absorption of Bi:YIG is taken into consideration and gray line assumes the MO material is lossless (no imaginary part in the refractive index).

To have a comprehensive study on MPCs, we start with a one-defect MPC. Figure 2 shows the transmission spectrum for the MPC of (H/L)^p^/(D/L)^q^/(H/L)^p^ structure (H: SiO_2_, L: TiO_2_, D: Bi:YIG) where p = q = 1 and thicknesses are set for green band. A band gap covering the visible range is observed including a transmission peak at λ = 519 nm.

The calculations are done with (black line) and without (gray line) material absorption of Bi:YIG, to observe the effect of its optical absorption on the transmission spectra of the MPC. The results reveal that the band gap caused by photonic crystal structure starts at a wavelength around 360 nm but due to considerable material loss in ultraviolet region, band edge is not discernible for the simulations where real material properties are applied.

**Figure 3:**
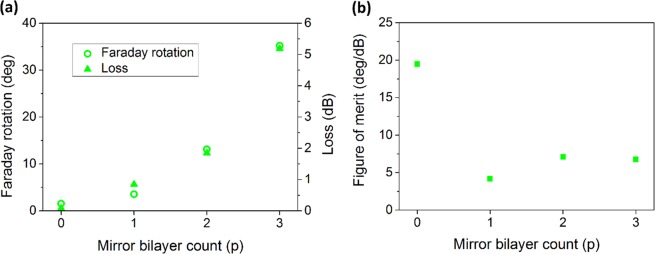
Effect of cavity mirror count on Faraday rotation and the MO figure-of-merit of the one-defect MPC. (**a**) Faraday rotation, loss and (**b**) figure of merit calculated for the one-defect MPC with (H/L)^p^/(D/L)/(H/L)^p^ structure, at its resonance peak around λ = 519 nm.

Figure 3 illustrates how number of (H/L) bilayers in Bragg mirrors changes performance of the MPC having a single defect. Both FR and loss increase by adding more mirror layers to the structure, but rise in loss dominates and FoM considerably drops in photonic crystal structure compared to the bare MO film (Fig. 3b).

### Two-defect MPC

**Figure 4:**
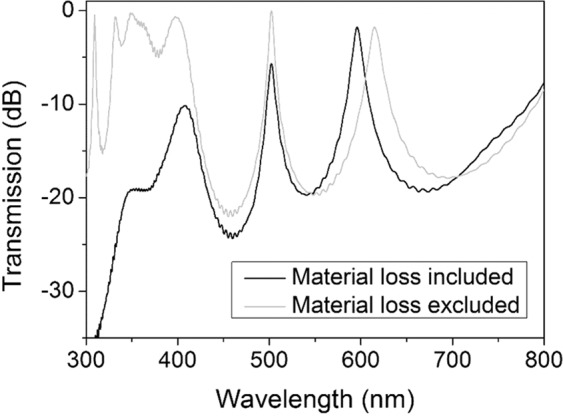
Transmission spectrum of the MPC with (H/L)^2^/(D/L)^2^/(H/L)^2^ structure. Black line shows the case where material absorption of Bi:YIG is taken into consideration and gray line assumes the MO material is lossless (no imaginary part in the refractive index).

Transmission spectrum of a two-defect MPC where each defect is accompanied by a set of H/L bilayer in the mirrors (p = q = 2) is depicted in Fig. 4. Adding a defect to the structure of MPC, not only causes appearance of a new transmission peak in the band gap but also changes the position of the first peak, as also observed previously^62^. Although the band edge in shorter wavelengths has slightly redshifted, the band gap still covers the visible range. Transmission peaks are at λ = 502 nm (green) and 596 nm (red).

**Figure 5:**
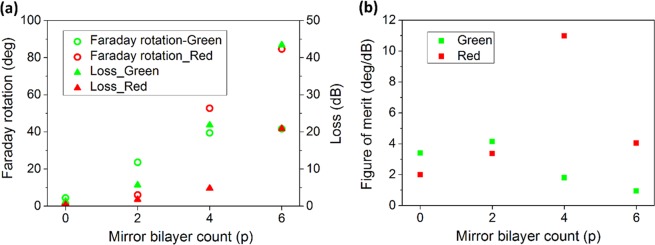
Effect of cavity mirror count on Faraday rotation and the MO figure-of-merit of the two-defect MPC. (**a**) Faraday rotation, loss and (**b**) figure of merit calculated for the two-defect MPC with (H/L)^p^/(D/L)^2^/(H/L)^p^ structure, at its resonance peaks around λ = 502 nm (green) and λ = 596 nm (red). Green (red) dots indicate Faraday rotation, loss and FoM for green (red) wavelength.

Figure 5 indicates how FR, loss and FoM for two resonance peaks of the two-defect MPC change by number of mirror bilayers in the structure. Increasing number of H/L layers notably improves FR such that with 6 repetition of bilayers in the mirrors, about 41° and 84° of rotation is achieved respectively for the peaks in green and red band. Progressively increasing loss for higher number of mirror layers reduces figure of merit for green color while for red, having four bilayers offers the optimum structure and leads to a FoM higher than 10°·dB^−1^.

### Three-defect MPC

**Figure 6:**
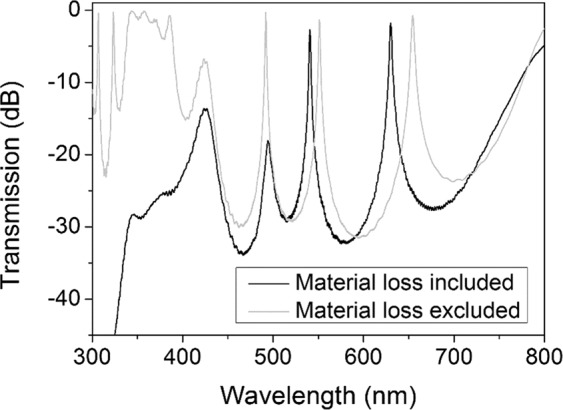
Transmission spectrum of the MPC with (H/L)^3^/(D/L)^3^/(H/L)^3^ structure. Black (gray) line shows the case where the MO material is lossy (lossless).

For an MOSLM which can perform modulation in RGB, we propose a magnetophotonic crystal with three defects presenting resonance peaks in red, green and blue band. Figure 6 demonstrates transmission spectrum for such an MPC in the form of (H/L)^3^/(D/L)^3^/(H/L)^3^. Layer thicknesses are optimized in a way that peak positions match RGB bands while retaining the FR at maximum and loss at minimum for each peak. H, L and D have thicknesses in the order of 50, 100 and 110 nm, respectively, giving an overall thickness less than 1.5 µm. By reducing the thickness of MO layers needed, we can also reduce the total energy required for switching each pixel. The band gap starts from the wavelengths around 425 nm and covers the rest of visible range. Transmission peaks for this MPC appear at λ = 494 nm (blue), 541 nm (green) and 630 nm (red). The obtained RGB wavelengths yield a good coverage across the chromaticity chart for a full color display. The comparison between two graphs in the figure indicates that in contrast to green and red regions, material loss is more substantial in blue, which causes a peak with considerably lower transmission. This issue needs to be addressed by synthesizing MO materials with high FoM and low loss in ideal RGB ranges.

**Figure 7:**
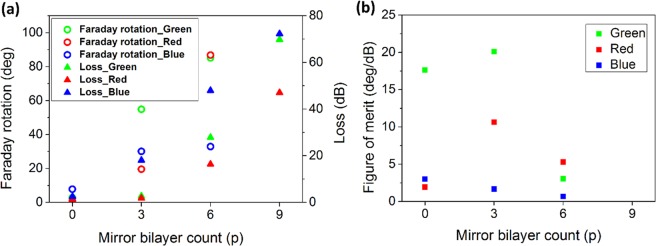
Effect of cavity mirror count on Faraday rotation and the MO figure-of-merit of the three-defect MPC. (**a**) Faraday rotation, loss and (**b**) figure of merit calculated for the three-defect MPC with (H/L)^p^/(D/L)^3^/(H/L)^p^ structure, at its resonance peaks around λ = 494 nm (blue), λ = 541 nm (green) and λ = 630 nm (red).

Figure 7 shows how performance of the three-defect MPC changes for different number of mirror layers. Rotations higher than 80° are achievable by 6 bilayers. Due to the trade-off between Faraday rotation and transmission, the structure with 3 H/L layers in the mirrors seems to be the optimal choice, as it has FoM higher than 10°.dB^−1^ in green and red band. This optimized structure shows FR of 20°, 55° and 30° for RGB, leading to the contrast values of Δ = tan(20)/tan(0.086) = 242.49, tan(55)/tan(0.086) = 951.47 and tan(30)/tan(0.086) = 384.65 in red, green and blue, respectively. By iteratively reducing this angle, one can achieve higher contrasts.

**Figure 8:**
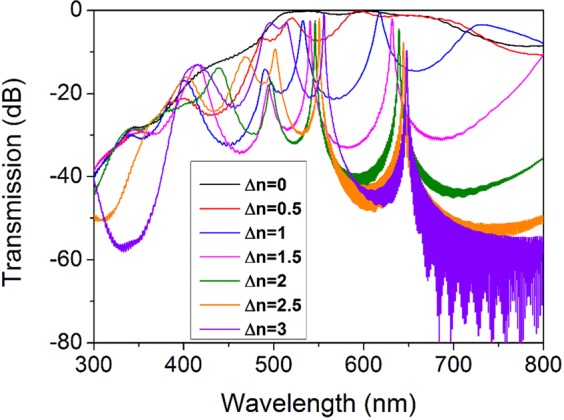
Transmission spectra of the three-defect MPC for different values of refractive index contrast between high- and low-index layers, Δn. No index contrast means no localization and increased index contrast slightly redshifts and sharpens the transmission peaks.

Although structural configuration plays the most important role in the performance of a photonic crystal, other factors such as index contrast between periodic dielectrics and layer thicknesses are also important^63^. Transmission spectra for MPCs of the same structure but having different index contrast between high- and low-index layers is shown in Fig. 8. Band gap and transmission peaks are not evident for the case of zero difference in refractive indices and emerge only by introducing index contrast to the system. Increasing Δn leads to sharper peaks in deeper band gaps. Moreover, broadening of the band gap and a shift to the longer wavelengths is observed.

**Figure 9:**
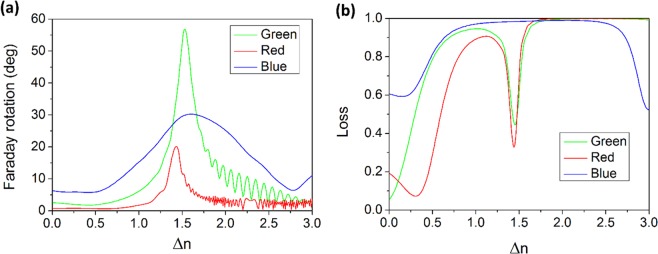
Effect of refractive index contrast of photonic crystal cavity layers on Faraday rotation and optical loss on red, green and blue wavelength outputs. (**a**) Faraday rotation and (**b**) loss of the three-defect MPC as a function of difference between refractive indices of high- and low-index layers. Blue, green and red curves represent λ = 494, 541 and 630 nm, respectively.

Figure 9 shows FR and loss of the three-defect MPC as a function of index contrast between H and L layers, for obtained RGB wavelengths of 494, 541 and 630 nm. By increasing Δn we expect both FR and loss to increase as a result of higher localization. Nevertheless, a peak is observed around Δn = 1.5 because our proposed MPC is constituted of TiO_2_ and SiO_2_ which have an index difference about 1.5 in the intended wavelengths. So the thicknesses are designed in a way that for such a Δn, resonance peaks of transmission and accordingly FR happen in the mentioned wavelengths. Oscillations in FR results for higher Δn is due to the high loss where E-field components used for calculation of FR become very small and highly sensitive to calculation errors. As can be seen from Fig. 8, for Δn values larger than 1.5, λ = 541 and 630 nm completely locate at dips of transmission spectra which causes the high loss level for green and red graphs in Fig. 9b. At λ = 494, however, for Δn > 2 band edge approaches the mentioned wavelength explaining reduction of loss for blue graph in Fig. 9b.

**Figure 10:**
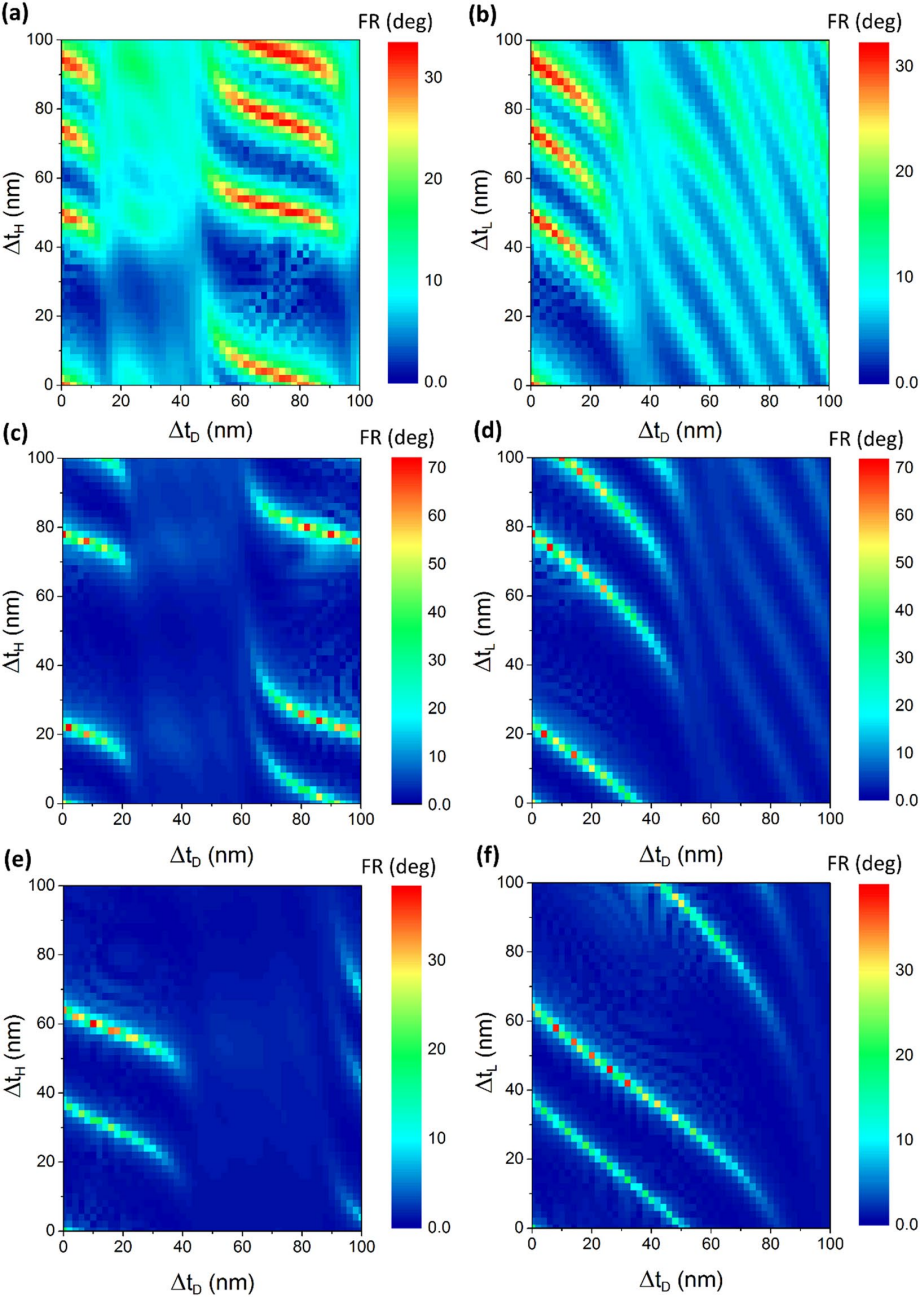
Effect of layer thicknesses on Faraday rotations at RGB wavelengths. Faraday rotation as a function of variation in thicknesses of layers from the primarily designed thicknesses for (**a**) and (**b**) λ = 494 nm, (**c**) and (**d**) λ = 541 nm, (**e**) and (**f**) λ = 630 nm. Δt_H_, Δt_L_ and Δt_D_ denote changes in thickness of high-index, low-index and defect layers, respectively.

Faraday rotation for RGB wavelengths as a function of layer thicknesses is demonstrated in Fig. 10. Cases with high FR are observed due to phase matching conditions and stronger localization in MO defects. Although specific FR for Bi:YIG material decreases with wavelength, the highest FR is achieved for green (Fig. 10c,d) implying that the maximum localization happens for this wavelength. Higher FR is achievable for red (Fig. 10e,f) compared to blue (Fig. 10a,b) in spite of its lower specific FR, which is indication of better localization in red than in blue. Since Bi:YIG has a higher specific FR in blue band and lower localization as discussed, even in the thickness combinations that localization is not happening efficiently, FR is comparable to the maximum value while in green and red there is a large difference between parts representing high localization and the unlocalized regions. Moreover, high-FR parts in red and green are much narrower than in blue, which shows higher sensitivity to thickness in these bands. Another point observable from Fig. 10 is that the thickness combination which we have chosen for the three-defect MPC, shown with point (0,0) in the color maps, is in high-FR conditions for all three wavelengths. In the optimized cases, there are a few nm thickness tolerances for each layer to retain high FR in the same wavelength.

**Figure 11:**
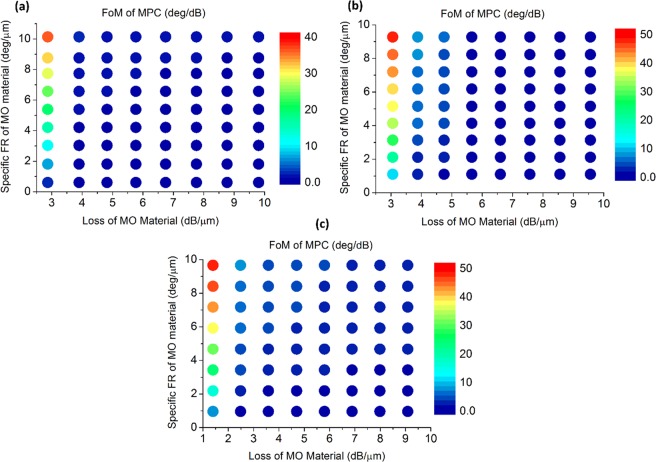
Effect of MO material FoM on MOSLM performance. Figure of merit for the three-defect MPC as a function of specific Faraday rotation and loss of the MO material, at the resonance peak around (**a**) λ = 494 nm, (**b**) λ = 541 nm and (**c**) λ = 630 nm. Color scales on the right of each figure indicates the figure of merit of MPC in °.dB^−1^.

Figure 11 shows how figure of merit for MPC changes as a function of specific Faraday rotation and loss of MO material. As can be seen from the color maps, FoM of MPC is more sensitive to loss of the material than its specific FR, i.e. by increasing FR of material, FoM for MPC increases gradually while increasing material loss from its minimum (caused mostly due to reflection) drastically decreases FoM. Maximum FoM attainable with the proposed three-defect MPC assuming an MO material with about 10°.µm^−1^ specific FR and almost zero absorption is lower for blue (Fig. 11a) compared to the other wavelengths. This is due to the weaker localization happening at this wavelength as discussed before. Green (Fig. 11b) and red (Fig. 11c) show maximum FoM in the same order while it is notable that reflection loss for red (about 1.5 dB) is lower than that for green (3 dB). Thus, one can conclude that compared to blue (Fig. 11a), higher FoM achievable in green (Fig. 11b) is due to better field localization and FR enhancement of the designed MPC at this wavelength, as minimum MO material losses for blue and green are almost the same. However, in the comparison of blue (Fig. 11a) and red (Fig. 11c), we can say higher FOM achievable in red is because the minimum loss of the MO material in red is lower than in blue.

## Conclusion

MOSLMs can be functional optical memory devices that can help control the phase and spatial distribution of light. Their intrinsic switching timescales allow for ultrahigh frequency operation in principle. In addition, recently discovered switching methods may enable researchers to revisit MOSLM structures for current or voltage control of magnetization and spatial light modulation. In this study, we indicate a few key design rules for MOSLM in the visible band and demonstrate a three-defect MPC-MOSLM capable of simultaneously enhancing Faraday rotation, and high-contrast modulation at three fundamental wavelengths of RGB within the same pixel. After optimization of configuration and layer thicknesses, an MPC structure with high Faraday rotation (20°, 55° and 30° for RGB, respectively) and low loss (2, 3 and 18 dB for RGB, respectively) with an overall thickness of 1.5 µm was presented. These rotation values become double by magnetization reversal, so the aforementioned structure enables modulation with 2θ_F_ = 40°, 110° and 60° of rotation for RGB, respectively. By keeping the MO layers as thin as possible, the required MO layer volume for magnetooptical switching would be lower. Thus, lower switching energy per pixel in MOSLMs can be achieved. Some of the key design rules for high FoM MOSLMs include:

1. Magnetophotonic crystal structures must use low-loss materials with large enough refractive index contrast for mirror layers (e.g. SiO_2_ and TiO_2_).
2. The magnetooptical FoM of the MO material needs to be maximized. In particular, at least a few degrees of Faraday rotation per dB optical loss is suggested. This is important for high performance MOSLM devices. The absolute value of loss of the MO materials should also be low enough (less than 2–3 dB·µm^−1^) in order for MOSLM devices to minimize absorptive optical losses (i.e. heating). In order to achieve acceptable signal-to-noise ratio at the output signal having more than 20° overall rotation is recommended for MOSLM.
3. The mirror layer count needs to be optimized such that optical losses can be kept below 10 or at most 20 dB, while sufficiently many pairs of layers should be in place to ensure Faraday rotation enhancement.
4. Faraday rotation is maximized when index and phase matching conditions are satisfied for the cavity layers with a given index contrast Δn.
5. Since photonic crystal defect modes hybridize, multi-defect configurations need numerical optimization. Examples of such optimizations are provided in Figs 4–7.
6. One needs to pay special attention to the layer thickness tolerances as shown on Fig. 10. While Faraday rotation is not significantly affected in blue as long as the layer thicknesses are within ±5% of the target thickness, the tolerances are limited by the red wavelengths, where the tolerance for layer thicknesses is below ±2%. Therefore, significant cleanliness and deposition process control in multilayer fabrication is essential.
7. The MOSLM device packaging must include two polarizers (one at the input, the other at the output) at perfectly perpendicular orientations to achieve extinction condition. This allows the contrast to be arbitrarily scalable from Δ = tan(θ_F_)/tan(θ_p_) = tan(θ_F_)/tan(0.086) = tan(θ_F_) · 666.23. An improved precision over the control of polarizer orientations relaxes the Faraday rotation requirements on the MO defect materials.

Following these criteria, magnetophotonic crystals can significantly enhance Faraday rotation in the visible range, realizing MOSLMs capable of efficient modulation in fundamental red, green and blue bands suitable for a full-color display system. We showed that bismuth-substituted yttrium iron garnets are promising material candidates for this purpose and characterized different MPCs of the form (H/L)^p^/(D/L)^q^/(H/L)^p^. We demonstrated that in addition to RGB modulation, the three-defect MPC provides better performance in terms of achieved FR and FoM. Faraday rotations higher than 20° and figures of merit up to 20°·dB^−1^ were achieved. The parametric evaluation done to investigate how performance of such an MPC changes as a function of structural variations and material properties indicates that layer thicknesses and material losses need to be well controlled. Development of new MO materials with high structural stability, high Faraday rotation and low optical loss (high figure-of-merit) would help develop new high-performance MOSLM devices.

## Methods

Lumerical FDTD Solution which is a Maxwell’s equation solver based on finite-difference time-domain method has been used to calculate the field components and obtain transmission and FR values. Linearly polarized plane wave with normal incidence was used as light source in the simulations, and applied boundary conditions were absorbing in the propagation direction and periodic in the other directions.

MO material was defined by its permittivity tensor including off-diagonal elements which are responsible for MO activity:

$$\begin{array}{ccc}{\rm{\varepsilon }} & = & (\begin{array}{ccc}{{\rm{\varepsilon }}}_{{\rm{1}}} & {+i{\rm{\varepsilon }}}_{{\rm{2}}} & {\rm{0}}\\ -{i{\rm{\varepsilon }}}_{{\rm{2}}} & {{\rm{\varepsilon }}}_{{\rm{1}}} & {\rm{0}}\\ {\rm{0}} & {\rm{0}} & {{\rm{\varepsilon }}}_{{\rm{1}}}\end{array})\end{array}$$

Generally, ε_1_ and ε_2_ have complex values and both their real and imaginary parts are dispersive with respect to wavelength. We have taken the values for Bi_1_Y_2_Fe_5_O_12_ from ref. ^52^.
